# Guava leaf extracts alleviate fatty liver via expression of adiponectin receptors in SHRSP.Z-Leprfa/Izm rats

**DOI:** 10.1186/1743-7075-9-13

**Published:** 2012-02-20

**Authors:** Hisae Yoshitomi, Xiangyu Guo, Tonghua Liu, Ming Gao

**Affiliations:** 1 School of Pharmaceutical Sciences, Mukogawa Women's University, Nishinomiya, Japan; 2 Beijing University of Chinese Medicine subsidiary Dongfang Hospital, Beijing, China; 3 Beijing University of Chinese Medicine, Beijing, China; 4 11-68 Koshien Kyuban-cho, Nishinomiya 663-8179, Japan

**Keywords:** Fatty liver, Adiponectin, Guava leaf extract, SHRSP.Z-Leprfa/IzmDmcr rat

## Abstract

**Background:**

In recent years, the number of people with metabolic syndrome has continued to rise because of changing eating habits, and accompanying hepatic steatosis patients have also increased. This study examined the effect of guava leaf extract on liver fat accumulation using SHRSP.Z-Leprfa/IzmDmcr rats (SHRSP/ZF), which are a metabolic syndrome model animal.

**Method:**

Seven-week-old male SHRSP/ZF rats were divided into two groups, a control group and a guava leaf extract (GLE) group. We gave 2 g/kg/day GLE or water by forced administration for 6 weeks. After the experimental period, the rats were sacrificed and organ weight, hepatic lipids, serum aminotransferase and liver pathology were examined. To search for a possible mechanism, we examined the changes of key enzyme and transcriptional factors involved in hepatic fatty acid beta-oxidation.

**Results:**

The triglyceride content of the liver significantly decreased in the GLE group in comparison with the control group, and decreased fat-drop formation in the liver tissue graft in the GLE group was observed. In addition, the improvement of liver organization impairments with fat accumulation restriction was suggested because blood AST and ALT in the GLE group significantly decreased. Furthermore, it was supposed that the activity of AMPK and PPARα significantly increased in the GLE group via the increase of adiponectin receptors. These were thought to be associated with the decrease of the triglyceride content in the liver because AMPK and PPARα in liver tissue control energy metabolism or lipid composition. On the other hand, insulin resistance was suggested to have improved by the fatty liver improvement in GLE.

**Conclusion:**

Our results indicate that administration of GLE may have preventive effects of hepatic accumulation and ameliorated hepatic insulin resistance by enhancing the adiponectin beta-oxidation system. Guava leaf may be potentially useful for hepatic steatosis without the side effects of long-term treatments.

## Background

In recent years, metabolic syndrome has attracted attention worldwide as it concerns type II diabetes, hyperlipidemia and hypertension developing against the background of visceral obesity, and the pathogenic elucidation of metabolic syndrome has been pushed forward. Accumulation of excessive amounts of storage lipids into adipose tissue causes adipocyte hypertrophy with the development of obesity, subsequently resulting in secretion abnormalities of FFA and adipocytokines such as TNF-alpha, IL-6 and adiponectin derived from adipocytes, which are involved in insulin resistance [1]. This is the source of metabolic syndrome.

Hepatic steatosis, which develops when a large amount of lipid is deposited in hepatocyte is the liver manifestation of metabolic syndrome [2]. It is a common liver disease and is rapidly becoming a worldwide public health problem [3]. Currently, there are no data on the long-term administration of drugs for hepatic steatosis and effective therapy for hepatic steatosis has not been established, so the treatment generally requires an improved lifestyle, such as reducing a high calorie intake and increasing exercise; however, many patients have poor compliance. Therefore, the discovery of nutrients that ameliorate fatty liver is of interest and value.

The guava tree (*psidium guajava *linn.) belongs to a member of the Myrtaceae family, which range throughout tropical and subtropical countries. It is commonly used not only as food but also as folk medicine as an antioxidant, anti-spasmodic, anti-allergy, anti-inflammatory and anti-diabetic using extracts from the fruit, leaf, bark or roots [4,5]. Leaves of this plant have been used as folk medicine and are reported to contain several compounds, such as various terpenoids [6,7], flavonoids [8] and tannins [9]. Previous reports demonstrated the effects of guava leaf extracts (GLE) against, for example, diabetes [10-12]; however, there have not yet been precise studies of the effects of GLE on hepatosteatosis.

SHRSP.Z-Leprfa/IzmDmcr rats (SHRSP/ZF) were established as a new model of metabolic syndrome by crossing SHRSP rats (this model presents with marked elevation of blood pressure) with Zucker Fatty rats. SHRSP/ZF rats undergo a mutation in the leptin receptor, and develop obesity, hyperlipidemia and insulin resistance [13]; thus, they are good tool for the study of metabolic syndrome-associated diabetes, hepatic steatosis and so on [14].

Recently, the search for appropriate hepatic steatosis agents has focused on plants used in traditional medicine. The present study investigated whether GLE exhibits preventive effects against hepatic accumulation and ameliorative hepatic insulin resistance, and the mechanisms behind such effects using SHRSP/ZF rats.

## Methods

### Animals

Six-week-old male SPF (specific pathogen free) SHRSP.Z-Leprfa/IzmDmcr (SHRSP/ZF) rats were obtained from Japan SLC (Shizuoka, Japan). All rats were housed in a climate-controlled (temperature: 22-24°C, humidity: 40-60%) light-regulated room with 12-hour light and dark cycles. The rats were fed normal chow (CE-2) for 1 week to stabilize their metabolic condition. SHRSP/ZF rats were allotted to 2 groups; a control group (not treated, n = 7) and a guava leaf 70% ethanol extract (GLE) group (GLE gavage administered as 2 g/kg/day, supplied by Beijing University of Chinese Medicine, which identified the biological materials, n = 7). We measured the water and food intake and body weight (BW) of rats daily.

At the conclusion of the 6-week treatment period, after 12 h fasting, all rats were sacrificed. Their blood samples were collected and sera were prepared by centrifugation, frozen, and stored at -20°C until analysis. The tissues were immediately harvested and cleaned for measurement of tissue weight, and the liver was promptly frozen in liquid nitrogen and stored at -80°C for Western blotting and gene analysis. A portion of the liver from individual rats was fixed overnight in 10% formalin for histological analysis.

All procedures were carried out in accordance with the guiding principles for the care and use of animals in the field of physiological sciences established by the Physiological Society of Japan, and the guidelines for the care and use of animals set by Mukogawa University.

### Blood analysis

The concentrations of serum aspartate aminotransferase (AST) and alanine aminotransferase (ALT) were measured using the corresponding commercial enzyme kit (Wako Pure Chemicals, Osaka).

### Tissue lipid analysis

Liver tissue (10 mg) was homogenized in 0.1 M acetic acid and hepatic lipid extracted with chloroform-methanol, as described by Folch *et al. *[15]. Chloroform layers were dried and dissolved in isopropanol containing 10% Triton X-100. Triglyceride, cholesterol and FFA concentrations were then determined enzymatically using each kit (Wako).

### Histological analysis of the liver

After fixation in 10% formalin buffer, liver tissue was embedded in paraffin. The liver sections were stained with hematoxylin and eosin (HE).

### Assay of hepatic CPT activity

A piece of liver was homogenized in 10 vol of 0.25 M sucrose, 1 mM EDTA and 3 mM Tris-HCl (pH7.2) solution buffer. After precipitating the nuclei fraction, the supernatant was used for enzyme activity. The activity of carnitine palmitoyl transferase (CPT) was determined as described previously [16]. The reaction solution contained 58 mM Tris-HCl (pH8.0), 1.25 mM EDTA, 0.25 mM 5, 5'-dithiobis-2-nitrobenzoic acid, 0.1% Triton-X and 0.04 mM palmitoyl-CoA. This solution was equilibrated at 30°C. The reaction was initiated by the addition of 0.1 mL supernatant and absorbance was monitored at 412 nm until it stabilized. After the addition of 1.25 mM L-carnitine, the rate was monitored and the difference between with and without L-carnitine gave the L-carnitine-dependent rate.

### Primary antibodies used

Immunoblotting was performed with the following commercially available antibodies; anti-rabbit AMPK, anti-rabbit phospho- AMPK, anti-rabbit Akt, anti-rabbit phospho-Akt (Ser473, Thr308), were from Cell Signaling Technology (Beverly, MA). Anti-mouse β-actin was obtained from Sigma (St. Louis, MO).

### Western blot analysis

The liver tissue was homogenized with ice-cold homogenized buffer containing 50 mM Tris-HCl (pH7.4), 100 mM NaCl, 1% Nonidet-P40, 0.25% Na deoxycholate, 0.1% SDS, 1 mM EDTA, 50 mM NaF, 2 mM Na_3_VO_4, _30 mM Na pyrophosphate, 2 mM PMSF, 1 mM benzamidine, 0.02 g/mL trypsin inhibitor, 0.02 g/mL leupeptin, and 0.02 g/mL aprotinin. After incubation for 2 hours, lysates were centrifuged at 15,000 rpm for 20 min and supernatants were isolated. Proteins were extracted by boiling the tissues in 0.5 mmol/l Tris/HCl, pH6.8, glycerol, 10% SDS, 0.1% bromophenol blue and 2-mercaptethanol. The proteins (25 μg/lean) were electrophoresed using 7.5%-12.5% SDS-PAGE gel at 100 V for 2 hours. After fractionating, the proteins were transferred onto a PVDF membrane (Amersham Life Science Inc. Buckinghamshire) at 100 mA for 2 hours. The membrane was blocked in Blocking One (Nacalai Tesque, Kyoto) for 20 min. After appropriate blocking, the blot was incubated with the primary antibody in antibody solution 1 (Toyobo, Osaka) overnight. It was then washed with TTBS and finally incubated for 1 h with a 1:5000 dilution of anti-rabbit and mouse IgG-horseradish peroxidase. Detection was achieved using an ECL kit (Amersham Inc. Buckinghamshire). β-actin was used as an internal control. The density of the bands was measured using NIH Image.

### Real-time RT PCR

Total RNA was extracted from the liver using Sepasol-RNA I Super G (Nacalai Tesque, Kyoto). From each sample, total RNA was reverse transcribed to cDNA using the ReverTra Ace qPCR RT Kit (Toyobo). THUNDERBIRD SYBR qPCR Mix was used for quantitative real-time RT-PCR analysis of each gene expression. The primers are listed in Table 1. Amplification was performed with a real-time PCR system (ABI Prism 7000). The results are expressed as a relative value after normalization to the β-actin expression.

**Table 1:** Primer sequences

Gene	Forward	Reverse
AdipoR1	TGAGGTACCAGCCAGATGTC	CGTGTCCGCTTCTCTGTTAC
AdipoR2	TCCATGGAGTCTCAAACCTG	GGAGAGTATCACAGCGCATC
PPAR-alpha	TGGAGTCCACGCATGTGAAG	TGTTCCGGTTCTTTTTCTGAATCT
MCAD	TGTGCCTACTGCGTGACAGA	TTCATCACCCTTCTTCTCTGCTT
ACO	CCCAAGACCCAAGAGTTCATTC	CACGGATAGGGACAACAAAGG
β-actin	GGGAAATCGTGCGTGACATT	GCGGCAGTGGCCATCTC

### Statistical analysis

Data are expressed as the mean ± SEM. Statistical analysis of the data was performed by Student's *t*-test. A *p*-value less than 0.05 was considered significant.

## Results

### Effect of GLE treatment on metabolic parameters

The metabolic parameters are shown in Table 2. After the 6-week treatment period, the body weight of the GLE group rats had significantly decreased compared with the control group (*p *= 0.027). BW gain was about 20% lower in the GLE group. Organ weight is also shown. Administration of GLE indicated a significant decrease in adipose weight (*p *= 0.007). In terms of liver weight reduction, liver TG was significantly reduced by GLE administration. On the other hand, GLE did not affect cholesterol and FFA in the liver. Meanwhile, daily food intake per cage did not differ among groups in either long-term experiment (data not shown).

**Table 2:** Effect of GLE on lipid parameters in SHRSP-fatty rats

	control	GLE
**Body Weight**	(n = 6)	(n = 5)
initial (g)	208.50 ± 9.12	216.40 ± 8.95
final (g)	405.17 ± 5.54	376.20 ± 10.04 *
gain (g)	196.67 ± 7.40	159.80 ± 17.05
**Organ Weight**	(n = 5)	(n = 4)
liver (g)	13.55 ± 0.56	12.39 ± 0.37
adipose (g)	9.76 ± 0.35	8.41 ± 0.32 *
**Liver lipid**	(n = 5)	(n = 4)
TG (mg/dL)	21.44 ± 1.42	12.85 ± 1.28 *
cholesterol (mg/dL)	26.78 ± 1.08	24.54 ± 0.76
FFA (mEq/L)	0.31 ± 0.01	0.38 ± 0.06

Data are the mean ± SEM. * *p *< 0.05 different from the control group. Rats were administered the treatment for 6 weeks

### Histological examination and serum aminotransferase

**Figure 1:**
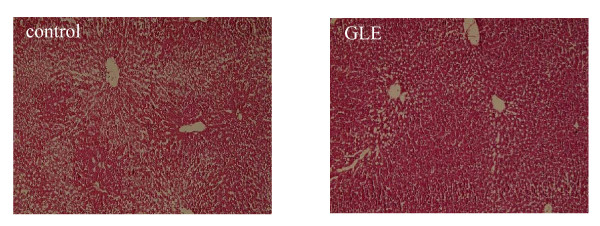
**Pathogenic description of the rat livers**. Effect of oral administration of GLE on the histological examination of livers from SHRSP-fatty rats. Liver tissue sections were stained with HE and examined under a light microscope. Data are representative images (magnification × 100).

The histological examination of livers from the experimental rats is shown in Figure 1. It can be seen that livers in the control group appeared to show a fatty liver. Administration of GLE showed a positive effect, and the GLE group improved and the liver appeared to be less fatty than the control group in the filtration of hepatocytes. This result was exemplified by the fact that levels of serum alanine transaminase (ALT) and aspartate transaminase (AST) in the GLE group were significantly lower than in the control group (Table 3: *p *= 0.019 and *p *= 0.049).

**Table 3:** Serum aminotransferase

	control (n = 5)	GLE (n = 4)
AST (IU/L)	47.23 ± 5.56	27.70 ± 1.31 *
ALT (IU/L)	24.37 ± 1.79	18.74 ± 1.41 *

Data are the mean ± SEM. * *p *< 0.05 different from the control group. Rats were administered the treatment for 6 weeks

### Changes in gene expression of adiponectin receptors

**Figure 2:**
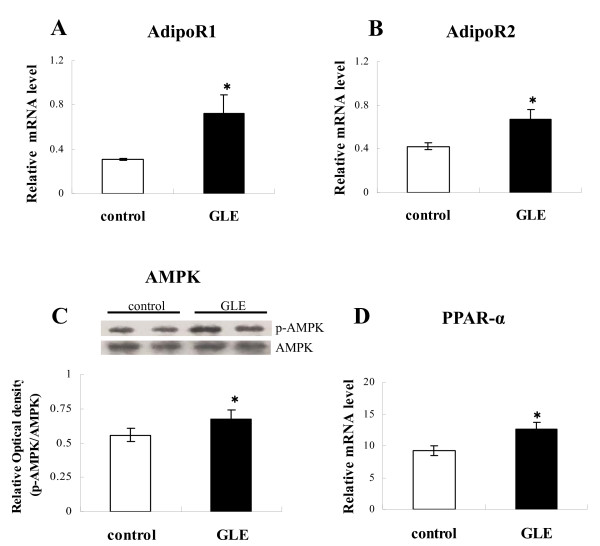
**Hepatic factors involved in energy metabolism in rats**. Effect of GLE on hepatic energy metabolism factors in rat liver: (**A**) AdipoR1 mRNA expression and (**B**) AdipoR2 mRNA expression (**C**) rate of AMPK phosphorylation, level of Western blotting (**D**) PPAR-alpha mRNA expression. Data are the mean ± SEM of n = 4 rats. * *p *< 0.05 different from the control group.

To elucidate the molecular mechanism of the lipid-lowering effect of GLE, we characterized adiponectin receptor gene expression in the liver. The results demonstrated significantly increased AdipoR1 (Figure 2A; 0.311 ± 0.011 vs 0.725 ± 0.166: *p *= 0.047 n = 4) and AdipoR2 (Figure 2B; 0.424 ± 0.034 ± vs 0.669 ± 0.094: *p *= 0.049 n = 4) in the GLE group compared with the control group.

### AMPK phosphorylation and PPARα expression

Adiponectin activity stimulates AMPK and PPARα, which are involved in liver lipid metabolism. Activity of AMPK was assessed by measuring phosphorylated AMPK (p-AMPK). Compared with the control, GLE-treated rats exhibited significantly increased p-AMPK protein levels (Figure 2C; 0.559 ± 0.047 vs 0.672 ± 0.071: *p *= 0.014 n = 4). Likewise, PPARα mRNA expression in the GLE group also became higher than in the control group (Figure 2D; 9.208 ± 0.722 vs 12.618 ± 1.073: *p *= 0.039 n = 4).

### Hepatic fatty acid beta-oxidation related enzyme expression and activity

**Figure 3:**
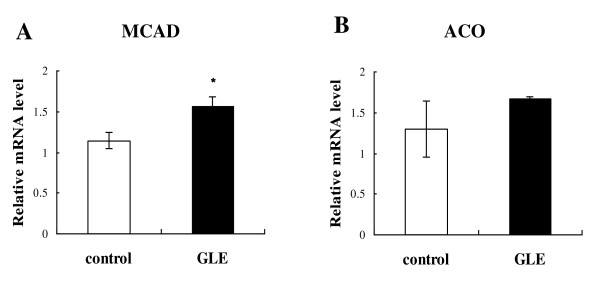
**Hepatic mRNA expression of enzymes involved in beta-oxidation in rats**. The expression of hepatic (**A**) MCAD and (**B**) ACO in rats treated GLE. Data are the mean ± SEM of n = 4 rats. * *p *< 0.05 different from the control group. Rats were administered the treatment for 6 weeks.

**Figure 4:**
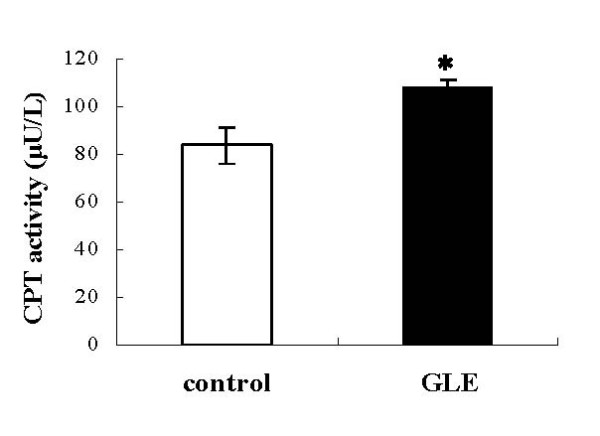
**Effect of GLE on hepatic CPT activity in rats**. Hepatic CPT activity in rats administered the control or GLE for 6 weeks. Data are the mean ± SEM of n = 4 rats. * *p *< 0.05 different from the control group.

We researched the expression of genes and activity involved in fatty acid beta-oxidation enzyme. First, we measured the expression genes, medium-chain acyl-CoA dehydrogenase (MCAD) and acyl-CoA oxidase (ACO), which are rate-limiting fatty acid beta-oxidation enzymes in mitochondria and peroxisome. In the GLE group, both were elevated compared with the control group (Figure 3A; 1.145 ± 0.094 vs 1.568 ± 0.117: *p *= 0.031 n = 4, and 3B; 1.296 ± 0.343vs 1.664 ± 0.036: *p *= 0.407 n = 4; differences were not statistically significant). Similarly, mitochondrial fatty acid beta-oxidation in the liver was increased after GLE administration, as measured using hepatic CPT activity (Figure 4; 83.65 ± 7.346 vs 107.65 ± 3.488 U/L: *p *= 0.03 n = 4).

### Effect of GLE treatment on Akt/PEPCK signaling in the liver

**Figure 5:**
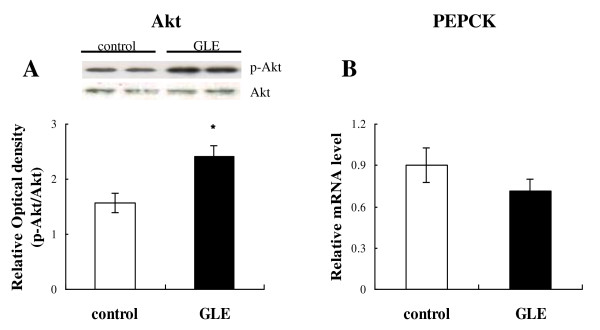
**Effect of GLE on hepatic Akt/PEPCK signaling in rats**. Effect of GLE on hepatic insulin signaling factors: (**A**) rate of Akt phosphorylation, level of (**B**) PEPCK mRNA expression in rat liver. Data are the mean ± SEM of n = 4 rats. * *p *< 0.05 different from the control group.

The level of phosphorylation of Akt at ser473 in the liver in the GLE group was significantly higher than in the control group (Figure 5A; 1.56 ± 0.178 vs 2.41 ± 0.194: *p *= 0.015 n = 4), as determined by Western blotting, because Akt is an important factor in insulin signaling and the control of gluconeogenesis. Moreover, the mRNA level of PEPCK, which is controlled by Akt activity, was also decreased compared with the control group (Figure 5B; 0.901 ± 0.125 vs 0.712 ± 0.085: *p *= 0.26 n = 4, differences were not statistically significant).

## Discussion

The our major findings of the present study are as follows: 1) The administration of GLE had preventive effects against hepatic lipid accumulation and ameliorated hepatic insulin resistance; 2) The mechanism for this action was the increase of β-oxidation by increasing adiponectin receptors.

SHRSP.Z-Leprfa/IzmDmcr rats (SHRSP/ZF rats) were established as a model of metabolic syndrome. Specifically, they showed alterations in lipogenesis, such as SCD-1, FAS and SREBP-1 in the liver. Abnormality of these genes caused the accumulation of lipid droplets in hepatocytes [17], and thus, they may be a good tool for the study of fatty liver. Using these model rats, we demonstrated that 6-week GLE treatment reduced body weight, liver weight and fat weight. The most interesting finding in our study was that GLE treatment ameliorated hepatic steatosis and decreased the accumulation of triglyceride (TG) in the liver of SHRSP/ZF rats. This was shown histologically and biochemically.

Alanine transaminase (ALT) is an important enzyme found predominately in the liver. Significantly elevated activity of ALT in serum often suggests its leakage from damaged hepatocytes and reflects hepatic damage. Aspartate aminotransferase (AST) is similar to ALT in that it is another enzyme associated with the liver [18]. A slight rise of AST and the ALT can be appeared for the fatty liver because of cellular membrane defect from FFA, and they are used as diagnostic criteria of the fatty liver. The decrease of serum ALT and AST by GLE also accounts for the improvement of the liver histology and less fat infiltration of hepatocytes.

In visceral obesity, adipocytokines such as TNFα, resistin, IL-6 and FFA are secreted by enlarged fat cells of the internal organs as adipose tissue and induce insulin resistance [19-21]. As a result of insulin resistance, glucose and fat metabolism decrease with the muscle, and the release of fatty acid is promoted with adipose tissue. Glucose and fatty acid are then supplied to the liver excessively. Furthermore, the biosynthesis of fatty acid is aggravated in the liver, and fat over-accumulates in hepatocytes in conjunction with fatty acid supplied from adipose tissue. On the other hand, β-oxidation, which is the fatty acid metabolism system in hepatocytes, decreases. As a result, a fatty liver develops.

Adiponectin is one such adipocytokine secreted by adipose tissue that has recently attracted much attention, because it has lipid metabolism promotion effects and improves insulin resistance [22]. In the liver, adiponectin stimulate glucose utilization and fatty acid combustion via AMPK. Adiponectin also increases the expression levels of PPARα and activates PPARα ligand, thereby stimulating the expression enzyme involved in fatty acid oxidation and decreasing tissue TG content [23]. Expression of adiponectin is induced by the differentiation of fat cells. In non-obese small fat cells, there is a high expression of adiponectin; however, its transcription is inhibited by obesity when a fat cell is enlarged. In our first approach to find a possible mechanism, we focused on adiponectin. We predicted that secretion of adiponectin might increase in the GLE group compared with the control group in rat adipose tissue from the results of decreased body weight and fat weight with GLE treatment; however, the serum adiponectin level did not increase with GLE treatment (data not shown). According to a past report, obesity decreases not only plasma adiponectin but also adiponectin receptor expression due to hyperinsulinemia [24]. We have already confirmed that SHRSP/ZF rat expression of adiponectin receptors is decreased compared with normal rats. In our study, GLE increased the expression of adiponectin receptor 1 and 2, with the activity of AMPK and mRNA expression of PPARα, which is a downstream adiponectin receptor.

Fatty acid β-oxidation takes place in two cellular organelles, mitochondria and peroxisomes [25]. In liver cells, it is proposed that mitochondrial oxidation might have the largest impact on total β-oxidation [26]. Activation of AMPK leads to a decrease in intracellular malonyl-CoA, which is a precursor of the biogenesis of fatty acid and a potent inhibitor of carnitine palmitoyltransferase (CPT) [27,28]. CPT is a rate-limiting enzyme of mitochondrial β-oxidation and plays an important role in the transport of fatty acid to inside the mitochondria [26]. On the other hand, PPARα controls the transcription of many genes involved in lipid catabolism, such as medium-chain acyl-CoA dehydrogenase (MCAD), acyl-CoA oxidase (ACO) and also CPT. In our study, it was demonstrated that CPT activity in the GLE group increased compared with the control. In addition, we evaluated mRNA expression; MCAD as a marker of enzyme mitochondrial β-oxidation, and ACO as a marker of point to control peroxisomal one [26]. In the GLE group, both were higher than in the control group. Our results showed that GLE treatment increases energy expenditure by increasing fatty acid β-oxidation in liver cells. On the other hand, SREBP-1 and FAS mRNA levels, which are factors in lipogenesis and downstream AMPK, showed no difference between them (data not shown).

Dysregulation of adiponectin and the decrease its receptors play causal roles in the development of insulin resistance [29]. A correlation has been reported that adiponectin receptor gene expression was decreased in type 2 diabetic patients. Moreover, a previous study demonstrated that AdipoR1/R2 knockout mice exhibited aggravated insulin resistance through increased TG content, inflammation and oxidative stress [30]. On the other hand, adipocyte hypertrophy with the development of obesity, subsequently resulting in secretion abnormalities of FFA and adipocytokines such as TNF-α and IL-6, which are involved in insulin resistance [1]. In this study, Akt activity in the liver in the GLE group was significantly higher than in the control group. Akt is an important factor in insulin signaling and in the control of gluconeogenesis. Moreover, the mRNA level of PEPCK, which is controlled by Akt activity and rate-limiting enzyme of gluconeogenesis, was also decreased compared with the control group. In our other group study, Guo et al. demonstrated that SHRSP/ZF rats treated with GLE had improvement glucose metabolism without enhanced insulin secretion. These results might suggest that GLE has an insulin resistance-improving effect by improving fatty liver and reduced adipose tissue due to a decreased adipocytokines.

## Conclusion

In conclusion, GLE increased adiponectin receptors in the liver and increased the activation of downstream AMPK and the expression of PPARα. Furthermore, it promoted fatty acid combustion by increasing the expression and activation of β-oxidative enzymes. It reduced TG content in the liver and, as a result, it improved insulin resistance and the inhibition of glyconeogenesis by synergistic interaction with AMPK. Recently, it was reported that fasting and exercise were factors increasing the expression of adiponectin receptors [31-33]; however, this is not easy, because they require a sustained effort and cause stress to patients. In addition, troglitazone and rosiglitazone, which are PPARγ agonists, also increased the expression of adiponectin receptors; however, they have risks when administered preventively and long term to patients with a previous condition such as metabolic syndrome because hepatotoxicity is a side effect [34,35]. From our present study, we suggest that guava leaf extract promotes fat combustion, restricts fat accumulation in the liver by increasing adiponectin receptor gene expression, and improves insulin resistance. Because there are few side effects of long-term treatment, guava leaf extract may be potentially useful for hepatic steatosis.

## Abbreviations

GLE: Guava leaf extract; TNF: Tumor necrosis factor; FFA: Free fatty acid; TG: Triglyceride; ALT: Alanine transaminase; AST: Aspartate aminotransferase; CPT: Carnitine palmitoyltransferase; MCAD: Medium-chain acyl-CoA dehydrogenase; ACO: Acyl-CoA oxidase.

## Competing interests

The authors declare that they have no competing interests.

## Authors' contributions

HY carried out the studies, acquired the data, performed the data analysis, and drafted and revised the manuscript. XG participated in the animal experiments. TL provided the original idea for the work and organized the study. GM was involved in the design and organization of the study, interpreted the results and revised the manuscript. All authors have read and approved the final manuscript.
